# Diagnostic techniques for inflammatory eye disease: past, present and future: a review

**DOI:** 10.1186/1471-2415-13-41

**Published:** 2013-08-08

**Authors:** Stephen C Teoh, Andrew D Dick

**Affiliations:** 1 National Healthcare Group Eye Institute, Tan Tock Seng Hospital, 11 Jalan Tan Tock Seng, Singapore 308433, Singapore; 2 Eagle Eye Centre, Mount Alvernia Hospital, 820 Thomson Road, Medical Centre Block B, #02-11/17, Singapore 574623, Singapore; 3 School of Clinical Sciences, University of Bristol, Bristol Eye Hospital, Lower Maudlin Street, BS1 2LX Bristol, UK; 4 National Institute of Health Research Biomedical Research Centre, Moorfields Eye Hospital NHS Foundation trust and UCL Institute of Ophthalmology, London, UK

**Keywords:** Diagnosis, Uveitis, Ocular inflammation, Hypersensitivity, Polymerase chain reaction, Immunoglobulin, Cytokines, Autoimmunity, Autoregulation

## Abstract

Investigations used to aid diagnosis and prognosticate outcomes in ocular inflammatory disorders are based on techniques that have evolved over the last two centuries have dramatically evolved with the advances in molecular biological and imaging technology. Our improved understanding of basic biological processes of infective drives of innate immunity bridging the engagement of adaptive immunity have formed techniques to tailor and develop assays, and deliver targeted treatment options. Diagnostic techniques are paramount to distinguish infective from non-infective intraocular inflammatory disease, particularly in atypical cases. The advances have enabled our ability to multiplex assay small amount of specimen quantities of intraocular samples including aqueous, vitreous or small tissue samples. Nevertheless to achieve diagnosis, techniques often require a range of assays from traditional hypersensitivity reactions and microbe specific immunoglobulin analysis to modern molecular techniques and cytokine analysis. Such approaches capitalise on the advantages of each technique, thereby improving the sensitivity and specificity of diagnoses. This review article highlights the development of laboratory diagnostic techniques for intraocular inflammatory disorders now readily available to assist in accurate identification of infective agents and appropriation of appropriate therapies as well as formulating patient stratification alongside clinical diagnoses into disease groups for clinical trials.

## Review

### Introduction

Intraocular inflammatory eye diseases though relatively uncommon remain an important cause of visual impairment. For example, uveitis is the third leading cause of blindness [1-3]. Broadly, the underlying aetiologies are divided into infective and non-infective (presumed autoimmune or autoinflammatory) causes. Since the late 20^th^ century, advances in molecular techniques have led not only to increasing our understanding of the pathogenetic mechanisms that are associated with various forms non-infectious uveitides, but also to improved refined, sensitive and specific diagnosis of infectious causes. Our understanding of the cellular and molecular pathways enabled in uveitis has led to the adoption of various immunosuppressive agents to overcome the burden of corticosteroid use, traditional and entrenched in uveitis practice. In a recent survey of treatment patterns of non-infectious uveitis by Ophthalmologists in the USA, it was found that up to 60% of patients were still treated with greater than 30mg of steroids for more than 1.5 years as maintenance therapy to control inflammation and the use of immunosuppressive therapy was only used in 12% of patients. 75% of physicians were not aware of treatment guidelines for uveitis [4]. Such guidelines are based on data and evidence that include, over time, the iterative bench-to-bedside translation and delivering clinical evidence for use of anti-metabolites [5-12] and calcineurin inhibitors [13-16]. More recently, progress in targeted therapy with biologics targeted against cytokines (e.g. anti-IL-1, anti-IL-6 and anti-TNF-α) [17-24], soluble mediators (e.g. interferons) [25,26], or cell surface molecules (e.g. Alemtuzumab and CTLA-4 Ig) [27] are showing great promise in the control of refractory non-infective uveitides. There remains the need to provide randomized controlled trial evidence to confirm their efficacy, some of which are on going. There are increasingly guidelines and algorithms being developed for immunosuppressive and immunomodulatory therapies for non-infectious uveitis by harnessing the increasing evidence being developed, in for example Behcet’s disease, and adoption by governments [28]. Arguably on the contrary, infective uveitides are still managed based on the clinician’s experience as such a clinical diagnosis is sometimes based on clinical signs and symptoms, supported by demographic information, morphology, laterality and clinical history. One clear example is cytomegalovirus retinitis in HIV [29]. However in practice with many cases, investigations are often necessary to elucidate and differentiate an aetiology and importantly to discriminate those that directly cause an infectious disease versus those evoking an inflammatory disease, such as latent tuberculosis (TB) [30].

In practice, determination of an underlying aetiology is a routine and important step in the assessment and evaluation of a uveitic patient. 40-86% of patients have an underlying cause ranging from infectious to autoimmune causes, whilst the rest remains classified as idiopathic when no apparent cause can be identified, but the condition responds to standard anti-inflammatory therapy [31]. Whilst anti-infective agents do not alter the course or outcome of autoimmune or non-infective uveitis, such therapy has no deleterious effects per se on the condition except that of prolonged and untreated non-infectious inflammation. Conversely, the use of anti-inflammatory and immunosuppressive agents in infective uveitides is potentially devastating. As such, differentiation is crucial and defining infectious versus non-infectious causes is vital from the outset. Given the advances in molecular and cellular pathology and diagnostic ability ranging from laboratory to radiological tests (including X-rays, computed tomography (CT) scans, magnetic resonance imaging (MRI) scans, positron emission tomography (PET) scans and nuclear imaging), we are more enabled to make such diagnoses. In this review, we will focus on the laboratory, blood and immunological tests, and these will be further discussed.

Infective uveitides vary in prevalence according to geographic regions. Uveitides that were previously ‘undiagnosed’, labeled and treated as ‘idiopathic’ are increasingly recognized as related to, or directly caused by an infective cause as a result of progress in diagnostic techniques. For example, cytomegalovirus detection in aqueous with resultant therapeutic responses to antiviral agents have led to improved therapeutic outcomes in hypertensive uveitic entities such as related syndromes for example, Posner-Schlossman syndrome [32-34]. Fuchs’ heterochromic iridocyclitis has also been linked to some herpes viruses and Rubella [35-38], and Tuberculosis-related intraocular inflammation has seen resurgence in diagnosis following the development of newer diagnostic techniques.

### Role of diagnostic tests in intraocular inflammation

Diagnostic tests in search for an aetiology in intraocular inflammatory diseases have always been controversial, mainly due to its history of suspected lack of specificity and sensitivity of assays. Such views have therefore led to the concept that the need for detecting infectious agents or underlying inflammatory disease, whether for clinical or research purposes, to deliver improved and more tailored diagnosis or understanding of mechanisms of inflammatory disease must be balanced against the cost of the investigations, the available resources of the treating centre, the utility of the tests employed (given potential lack of sensitivity of assays) and finally, and particularly so in acute circumstances, the time taken to obtain results. This is in contrast to performing tests for the overall systemic health of the individual prior to commencement of immunosuppression that can further compromise health. In a wider perspective, traditionally a “textbook” list of relatively untailored investigations remains costly and may not until recently, contribute to either diagnosis or change in management. A retrospective review of patients with various types of uveitis showed that abnormal values of complete blood counts, plasma viscosity / erythrocyte sedimentation rate (ESR) and VDRL / TPHA did not contribute to establishing an underlying cause of the uveitis [39]. A Canadian survey demonstrated that most routine tests performed for the investigation of anterior uveitis lack sensitivity and specificity and have low diagnostic yields [40]. In general, investigations are uncommonly performed for anterior uveitides alone except in specific circumstances e.g. chronic or recurrent disease, unresponsive or worsening with anti-inflammatory treatment or in hypertensive anterior uveitides. On the other hand, patients with intermediate and posterior uveitides, or those patients that present with systemic symptoms and manifestations are usually investigated with a panel of screening tests that comprise an autoimmune and infective screen that typically include syphilis and tuberculosis- two infections that have protean as well as overlapping ocular manifestations. Further investigations with blood tests, imaging, molecular diagnosis of aqueous or vitreous samples, or biopsy depend on the clinical presentation of the disease.

### Innate & adaptive immunity in infection

Infective pathogens incite inflammatory responses that form the basis of many diagnostic tests. The bodies’ natural non-specific antigen-independent innate immunity comprising leukocytes, macrophages and complement activation, interacts with the phylogenetically newer and antigen-specific adaptive immune system comprising T- and B-cells responses through complex interaction involving chemokines, cytokines and specialized cells including dendritic cells, NK cells and macrophages, in response to the infectious challenges. The measurement of these responses, both quantitatively and qualitatively, allows an assessment of the immune status of the individual. The characteristic granulomatous inflammatory response generated by the interaction of pathogens and the CD4+ Th1 cells via IFN-γ following antigen presentation has formed the basis of hypersensitivity tests such as the Mantoux test. Immunoglobulins generated by activated B-lymphocytes are routinely detected or measured that indicate temporal activity of an infection. Advances in technology have also enabled direct measurements of the different levels of cytokines and chemokines, the relative profiles and levels of which can be used as adjuncts in the diagnosis of various infections and inflammatory processes. The complex interactions between innate and adaptive immunity that is hitherto not fully illuminated, are kept in constant regulatory checks and balances by a system of chemical mediators to ensure efficient elimination of pathogens [41-43]. A dysfunctional innate and adaptive immune system on the other hand, can result in unregulated, inappropriate and detrimental immune inflammatory responses including autoimmunity, allergy, allograft rejection and shock [30].

### Improvements in diagnostic techniques

#### Introduction

Diagnostic techniques have evolved from direct observation of hypersensitivity reactions and analyses of immunoglobulins, to polymerase chain reactions and the modern measurements of cytokines. Despite the multitude of new tests and techniques, none of the tests are diagnostic and all are limited by its specificities and sensitivities, and should be interpreted in tandem with clinical assessment. As such, clinicians often use combination tests, harnessing the different strengths of the tests, to better improve the specificity and sensitivity of diagnosis in a rapid and accurate manner. This often involves a mix of traditional and newer assays.

#### Combination of traditional hypersensitivity tests and modern cytokine assays

Hypersensitivity responses, a technique that been in use for the last century, remain commonly used in combination with modern molecular techniques to assist in the diagnosis of ocular tuberculosis (TB). TB-related intraocular inflammation is well-known to present in a myriad of protean manifestations. Diagnosis has always been difficult as direct isolation and culture is usually unavailable [44]. The small tissue and fluid samples that can be feasibly obtained from ocular samples further limits the ability to detect the fastidious mycobacterium organisms. Moreover, TB-associated intraocular inflammation is also thought to be immune-mediated, due to reaction to mycobaterial proteins in latent tuberculosis, rather than direct infection [44-46]. This often poses a treatment dilemma between Ophthalmologists and Internists wherein treatment with anti-tuberculosis drugs in these patients with non culture/smear-proven patients are often discouraged. The classic cornerstone diagnostic test is the tuberculin skin test (TST) (Mantoux skin test) where tuberculin injected intradermally to produce a localized granulomatous inflammatory response through the interaction of macrophages and memory Th1 CD4 T-helper lymphocytes in a type IV hypersensitivity reaction. The most important limitation of TST is its inability to differentiate *M. tuberculosis* and non-tuberculous mycobacterial infections. Recent molecular technique advancements including polymerase chain reaction (PCR) and the use of cytokine analysis in the form of interferon gamma release assays (IGRAs) have been added to the armamentarium of diagnostic tests to increase the specificity and sensitivity of the diagnosis of TB-associated uveitis. IGRAs detect the ability of *Mycobacterium tuberculosis* antigens [early secretory antigen target 6 (ESAT-6) and culture filtrate protein 10 (CFP-10)] to stimulate host production of IFN-γ, and are superior to TST in distinguishing latent TB infections (LTBI) from non-tuberculous mycobacteria and BCG vaccination [47] as it points to exposure to specific tuberculous antigens [48]. These antigens distinguish M. tuberculosis from most other mycobacteria. Although IGRA has not yet been widely tested in subjects with non-tuberculous mycobacterial infection, *M. kansasii, M. szulgai, M. marinum,* and *M bovis* may also yield positive results, as they share some common antigens [49,50] However these assays cannot distinguish *latent* from *active* TB infection as positivity merely indicates an *exposure* to Mycobacterium tuberculosis. Likewise, a positive TST may not distinguish between active disease and atypical mycobacterial infection and a negative avian Mantoux test does not exclude the latter diagnoses [51]. There are numerous causes for false-positive and false-negative interpretations of the TST [52]. Even in patients with proven non-tuberculous mycobacterial lymphadenitis, standard TST is only positive in about 50% of cases [53]. Each assay, therefore, is limited by its own specificities and sensitivities. A meta-analysis by Diel *et al.* inferred that IGRAs are superior to TST in diagnosis of active TB [54]. Ang *et al.* however reported that TST was more sensitive than T-SPOT.TB (Oxford Immunotec Ltd, Abingdon, UK) but T-SPOT.TB was more specific for diagnosing TB-associated uveitis. However a combination of techniques involving TST and IGRA is 2.16 times more likely to diagnose TAU [55]. A combination of both TST and IGRA may be useful in distinguishing between tuberculous and non-tuberculous disease, as well as active and latent disesase. In 2007, Gupta synthesized the strengths of these methods and proposed that a diagnosis of ‘presumed’ ocular TB can be made with a consistent clinical presentation of a granulomatous ocular inflammation alongside a positive TST or IGRA and/or isolation of mycobacterial DNA from ocular fluids or tissue using PCR [44,56].

#### Combination of traditional immunoglobulin analysis and modern polymerase chain reactions

Immunoglobulin analysis and polymerase chain reactions (PCR) are also commonly combined in the study of intraocular infection. Serological assessment (viz. IgG / IgM) is especially useful in diseases that are not prevalent or less common in the specific population and demographics of the patient. Coupled with signs consistent and compatible with an infection, a positive plasma serology can be interpreted as evidence of an infectious agent in intraocular inflammation. The observation of pathogen-specific immunoglobulin isotype class switching from IgM to IgG in serum, modulated by cytokines including IFN-γ, IL-4, IL-5 and TGF-β, has been interpreted to be a sign of recent infection. A positive IgM generally indicates primary or recurrent infection, but may be negative in immunocompromised individuals. Whereas a positive IgG suggests seroconversion usually after 2–4 weeks in paired sera samples or, in the absence of IgM antibodies, is usually indicative of past infection [57]. Within the eye however, only IgG-class antibody production has been detected [58]. The observation that the amount of this pathogen-specific intraocular antibody was correlated with the degree of plasma infiltration within uveal tissue led to a further refinement with the Goldmann-Witmer coefficient (GWC) since the 1970s [59-62]. PCR, with its high specificity and ability to analyze small aliquots of samples, has also been used widely in the aetiological detection of infective pathogens, masquerade syndromes and malignancies from ocular fluids. However, small volumes of samples are an inherent limitation that can result in systematic errors and false negatives. On the other hand, its high sensitivity rates can result in false-positive results. To overcome these shortcomings, a combination of GWC with PCR has been proposed to increase the sensitivity and specificity of detection [63]. De Groot Mijnes reported a higher detection rate for herpes viruses and toxoplasma with GWC and PCR assessment [59], and Talabani *et al.* and Villard *et al.* also reported an increased sensitivity of 80-83% for the detection of toxoplasma infection with GWC or enzyme-linked immunosorbent assay (ELISA) and PCR assessment compared to 70-73% with either technique alone [64,65].

In active endogenous uveitis, elevated immunoglobulins have also been detected both from the sera as well as aqueous. Elevated IgG, IgM and IgA levels have been measured in acute anterior uveitis [66-68]. Likewise, elevated non-specific IgG and IgA from aqueous has also been detected. It has been proposed that the presence of IgA response suggests an environmental or infectious aetiology acting across a mucosal tissue. However the lack of pathogen-specificity do not support an infective pathogenesis. On the other hand, the presence of elevated IgG antibodies, especially the detection of anti-retinal IgG antibodies, reinforces an autoimmune pathogenesis in “idiopathic” and non-infectious posterior uveitides.

#### Identification of new pathogens with new and combination techniques

The use of modern PCR and traditional immunoglobulin analysis with GWC has also enabled the identification of pathogens in uveitic entities previously thought to be idiopathic. Using GWC techniques, Fuchs’ heterochromic iridocyclitis has been attributed to Rubella and herpes viruses [35-38]. With the PCR technique, numerous organisms have also been identified from ocular fluids and implicated in ocular inflammation including HTLV-1, rubella, Epstein-Barr virus, HHV-6, human parechovirus, dengue and chikungunya virus [69-71]. The development of advanced techniques such as dot hydridization and multiplex PCR has also improved the sensitivity and rate of detection of several organisms simultaneously while maintaining good sensitivity and specificity [72,73]. A subset of Posner-Schlossman syndrome (PSS) was found to be associated herpetic viruses especially cytomegalovirus (CMV). CMV anterior uveitis has since been recognized as a separate entity with a different clinical course and poorer prognosis compared to PSS, often with more relapses and requiring anti-viral therapy [32-34,74]. The use of GWC and PCR has thus improved our understanding of aetiology and has new bearings on our management of ocular inflammatory diseases.

HIV is an increasing worldwide epidemic and is still on the increase every year [75]. The profound systemic immunosuppression in AIDS and immune reconstitution following modern day anti-retroviral therapy (ART) has resulted in a plethora of ocular inflammatory manifestation ranging from infection to non-infectious immunogenic immune recovery uveitis [76-81]. Not infrequently, ophthalmic manifestations can be the first indicator of HIV disease in patients who have not been previously tested. Whilst routine HIV testing is unnecessary in the assessment of patients with uveitis, a high index of suspicion should be borne in mind in the workup of these patients as there are major implications on the subsequent management including morbidity and mortality risks in patients who are HIV-positive. Patients who should be tested include: 1) patients with known HIV risk factors and high-exposure risk, 2) severe or bilateral posterior uveitis, retinitis or choroiditis, 3) features consistent with CMV retinitis without other known underlying causes of immunocompromise or immunosuppression, [82] 4) concomitant sexually transmitted diseases e.g. syphilis, 5) tuberculosis, 6) suspected herpes zoster uveitis in a young patient < 50 years, and 7) history of constitutional symptoms and unexplained lymphadenopathy [83].

#### Role of combination techniques in masquerade syndromes

The use of combination tests to improve the ability to detect and diagnose is also widely used in masquerade syndromes and intraocular lymphoma, conditions that are notorious for difficult diagnosis. Current diagnostic tests include the use of cytopathological analysis [84,85], flow cytometry [86], PCR demonstrating monoclonality and IgH gene rearrangements [87], and cytokine analysis. The relative levels of IL-10 vs IL-6 have been utilized as an adjunct in the diagnosis of primary intraocular lymphoma. IL-10 is preferentially expressed by B-cell malignancies and acts on B-lymphocytes to stimulate antibody production. In contrast, IL-6 is a principle mediator in endogenous and infective uveitides. A ratio of IL-10 to IL-6 levels of greater than 1.0 in both diluted and undiluted vitreous samples may act as a diagnostic tool to confirm intraocular lymphoma [88-93]. Kimura *et al.* found a detection rate of 91.7% in patients with B-cell lymphoma with or without vitritis [94]. Ohta *et al.* also reported a statistically significant IL 10:IL 6 ratio in patients with primary intraocular lymphoma compared to patients with uveitis (p < 0.0001) [95]. However the use of cytokine analysis alone is controversial as there are still no definitive diagnostic standards for the use of cytokines in diagnoses. The preparation of vitreous samples for cytopathological analysis has also changed over time to improve yields from these limited specimens. Intzedy *et al.* reported that samples placed in saline or prepared fresh followed by paraffin embedding was able to yield positive diagnosis in all specimens and this has remained the ‘gold-standard’ in cytological assessment [96]. Coupland *et al.* subsequently proposed that samples for prolonged transport be fixated with HOPE solution (Herpes-glutamic acid buffer mediated Organic solvent Protection Effect) improved the quality of cytomorphology and immunocytology with reduced artefacts when compared to unfixed vitreous specimens [97].

Diagnostic tests and techniques have expanded significantly, and clinicians are relying on combinations of tests to increase the sensitivity of detecting an aetiology. Nevertheless all diagnostic tests have their limitations and should still be interpreted within the clinical context for consistency [91].

### Autoimmunity & autoregulation

#### The role of autoantigens

The role of autoantigens against various cellular components is well-described in connective tissue diseases including systemic lupus erythematosus (SLE), rheumatoid arthritis (RA) and Sjogren’s. Autoantibodies immunoglobulins have been commonly used in the supportive diagnosis of connective tissue diseases. Rheumatoid factor, an antibody the Fc portion of IgG, is most relevant in rheumatoid arthritis. Other common autoantibodies described include anti-nuclear antibodies (ANA), double-stranded DNA (dsDNA) in connective tissue diseases and systemic lupus erythematosus, and anti-nuclear cytoplamsmic antibodies (ANCA) in Wegener’s granulomatosis and polyarteritis nodosa, amongst many others. These have become common diagnostic tests used by uveitis specialists when ocular inflammation is the first or only presentation of an auotimmune disease. Putative uveitogenic retinal antigens inciting autoreactive lymphocytes directly or indirectly by antigenic mimicry, such as the soluble Ag (sAg) and interphotoreceptor retinoid-binding protein (IRBP) have also been proposed to be involved in idiopathic posterior segment inflammatory conditions although there has been no consistent finding [98-102]. More recently dysregulation of the innate immune system (autoinflammation) has been recognized to be the underlying mechanism for various genetic and multifactorial disorders including Blau syndrome and Behcet’s disease resulting in non-specific inflammatory changes due to overexpression of chemokines and cytokines including IL-1, IL-6 and TNF-α [30,103]. These biomarkers have been described in various intraocular inflammatory conditions and purported to deliver both diagnostic and prognostic uses. Li *et al.* suggested that the combination of elevated CXCL10 (>500 ng/mL), CXCL8 (>30 ng/mL) and CCL2 (>60 ng/mL) was a biomarker to distinguish PSS samples with or without presence of CMV [104]. Ang *et al.* found that patients with TB-associated uveitis showed higher levels of IL-6, IL-8, CXCL9 and IP10, and was significantly different from idiopathic uveitis and controls [46] whilst Abu El-Asra found a significant positive association with TAU and IFN-γ, IL-8, MIG and IP-10 suggesting an autoimmune disease rather than an active TB infection. Active TB infection was typically associated with increased concentrations of IL12, TNF-α and IFN-γ [105]. Lahmar *et al.* reported that IL-5 and IL-12 were specific for ocular toxoplasmosis, and granulocyte monocyte colony-stimulating factor (GM-CSF) and IL-1 were specific for viral uveitis [106]. Jayant *et al.* also demonstrated significant differences in HIV patients with and without CMV retinitis compared to controls, and this difference continues to persist even in clinically quiescent retinitis [107]. Although the use of cytokine and chemokine biomarkers show promise, they still lack true specificity and may represent a pro-inflammatory acute phase reactant. The levels of cytokines most likely represent a balance of type 1 and type 2 cytokines resulting in ocular inflammation and damage [108]. Another potential diagnostic use of cytokine biomarkers is in assessment of clinical resolution of ocular inflammation. Current clinical indicator of resolution is based on SUN criteria [109], but these clinical signs do not predict relapse or subclinical inflammation. Often there are no laboratory markers of relapse for ocular inflammatory conditions, and even in AIDS patients on ART, the use of the ‘classical’ CD4 count can *fail* as a biomarker of immune recovery to predict control and suppression of CMV retinitis infection [110,111]. Cytokine and chemokine markers in these cases *may* prove to be useful diagnostic tools. As such further work is required to demonstrate validity of such relatively non-specific biomarkers or signatures for disease types when used either alone or in combination, for translation into clinical use.

#### Role of genetic factors

Genetic and environmental factors are also described in the interaction with autoimmunity. Ocular autoimmune disorders have been described to have a MHC class II or I association, mediating its effects through autoantigens or cross-reactivity to the MHC motifs from infectious antigens [101]. Seronegative arthropathies have been associated with HLA B27, whilst Birdshot chorioretinpoathy has been associated with HLA A29 [112-114], and a HLA-B*51-restricted peptide from an MHC class-I chain-related gene antigen has been shown to activate CD8+ T-cells with an up-regulated IFN-γ response in Behcet’s disease [115,116]. Although PCR analysis for HLA typing has thus been analyzed for pathological associations in ophthalmic disease [117], the use of HLA-typing for diagnosis is limited and should be interpreted with caution except B27 in recurrent anterior uveitis in undiagnosed or misdiagnosed spondyloarthropathies [118]. However, in complex intraocular inflammatory diseases that pose a diagnostic dilemma, a suggestive HLA typing may be of value in realigning our differentials. The role of HLA B27 may also have limited use in prognostication of anterior uveitis. Accoriniti *et al.* reported a higher incidence of systemic disease (p < 0.001) and 20% required immunosuppressive therapy [119]. Park *et al.* also reported a higher incidence of severe anterior chamber activity (p = 0.006), hypopyon (p = 0.034) and a higher frequency of recurrence (p = 0.007) [120].

## Conclusions

Diagnostic techniques in intraocular inflammation are constantly developing both technologically and through our advancements in our understanding of immunological processes involved. What has followed is development of such assays that are increasingly specific and sensitive to various pathologies (Table 1). However, despite the advancements, the clinical practice has to accept inherent limitations, and manoeuvre between the false-positives and false-negatives of each test and interpret results within clinical context. Nevertheless, with this armamentarium of assays and an appropriate utilisation of combination of these techniques, the uveitis specialist can move toward more accurate and early infectious-aetiological diagnosis and toward improving prognosis of intraocular inflammation as well as increased categorisation and stratification of patients to enable more focused clinical trials.

**Table 1:** An overview of validity of various tests (and combinations thereof) used in the diagnosis of infective uveitides

	**Infective agent**	**Assay**	***n (patients in studies)***	Validity*	**Reference**
*Ruokuonen et al.*	Rubella (in FHI)	Aqueous IgG	63	100.0%	[35]
		Aqueous PCR	20	10.0%	
*Suzuki et al.*	Rubella (in FHI)	GWC (> 3)	14	71.4%	[36]
		Aqueous PCR	9	22.2%	
*Quentin et al.*	Rubella (in FHI)	AI (≥ 1.5)	52	100.0%	[37]
		Aqueous PCR	28	17.9%	
*Ang et al.*	TB	IGRA (T-SPOT.TB)	162	Sp 75.0%, Sen 36.0%	[48]
		TST		Sp 51.1%, Sen 72.0%	
		TST + T-SPOT.TB		OR 2.16 (95% CI, 1.23-3.80)	
*De Groot-Mijnes et al.*	HSV	PCR / GWC +	13	46.2%	[50]
		GWC +		46.2%	
	VZV	PCR / GWC +	16	62.5%	
		GWC +		25.0%	
	Toxoplasma	PCR / GWC +	25	28.0%	
		GWC +		64.0%	
*Kiljstra et al. / Rothova et al.*	Toxoplasma	GWC	22-30	72.7%-93.3%	[53,54]
*Talabani et al.*	Toxoplasma	PCR + immunoblotting	54	Sen 73%	[55]
		GWC + immunoblotting		Sen 70%	
		PCR + GWC		Sen 80%	
		PCR + GWC + immunoblotting		Sen 85%	
*Villard et al.*	Toxoplasma	ELISA	19	Sp 85%	[56]
		Immunoblotting	19	Sp 85%	
		PCR	18	Sp 100%	
*Dabil et al.*	CMV, VZV, HSV, Toxoplasma	Multiplex PCR	21	85.7%	[61]
		Multiplex PCR		71.4% (loss of <1 log sensitivity)	

*Most studies are cohort studies and do not represent robust outcomes of validation.

Values stated are positive rates of detection unless otherwise specified.

*FHI* Fuchs’ heterochromic iridocyclitis, *GWC* Goldmann-Witmer Coefficient, *PCR* polymerase chain reaction, *AI* antibody index, *TB* Tuberculosis, *IGRA* interferon-gamma release assays, *TST* tuberculin skin test, *Sp* specificity, *Sen* sensitivity, *OR* odds ratio, *HSV* herpes simplex virus, *VZV* varicella zoster virus.

## Competing interests

The authors declare that they have no conflict of interest.

## Authors’ contributions

Both ADD and SCT contributed equally to the concept, preparation and editing of the manuscript. Both authors read and approved the final manuscript.

## Pre-publication history

The pre-publication history for this paper can be accessed here:

http://www.biomedcentral.com/1471-2415/13/41/prepub
